# The duck hepatitis virus 5'-UTR possesses HCV-like IRES activity that is independent of eIF4F complex and modulated by downstream coding sequences

**DOI:** 10.1186/1743-422X-8-147

**Published:** 2011-03-31

**Authors:** Guangqing Liu, Emilio Yángüez, Zongyan Chen, Chuanfeng Li

**Affiliations:** 1 Shanghai Veterinary Research Institute, Chinese Academy of Agricultural Sciences, Shanghai, 200241, PR China; 2 National Key Laboratory of Veterinary Biotechnology, Herbin, 150001, PR China; 3 Centro Nacional de Biotecnología, CSIC Darwin 3, Cantoblanco 28049, Madrid, Spain

## Abstract

Duck hepatitis virus (DHV-1) is a worldwide distributed picornavirus that causes acute and fatal disease in young ducklings. Recently, the complete genome of DHV-1 has been determined and comparative sequence analysis has shown that possesses the typical picornavirus organization but exhibits several unique features. For the first time, we provide evidence that the 626-nucleotide-long 5'-UTR of the DHV-1 genome contains an internal ribosome entry site (IRES) element that functions efficiently both *in vitro *and in mammalian cells. The prediction of the secondary structure of the DHV-1 IRES shows significant similarity to the hepatitis C virus (HCV) IRES. Moreover, similarly to HCV IRES, DHV-1 IRES can direct translation initiation in the absence of a functional eIF4F complex. We also demonstrate that the activity of the DHV-1 IRES is modulated by a viral coding sequence located downstream of the DHV-1 5'-UTR, which enhances DHV-1 IRES activity both *in vitro *and *in vivo*. Furthermore, mutational analysis of the predicted pseudo-knot structures at the 3'-end of the putative DHV-1 IRES supported the presence of conserved domains II and III and, as it has been previously described for other picornaviruses, these structures are essential for keeping the normal internal initiation of translation of DHV-1.

## Background

Duck hepatitis virus (DHV-1) is worldwide distributed and causes acute and fatal disease in young ducklings with severe economic impact in poultry industry. Although the disease was firstly reported in Long Island in 1949 [1], the complete genome of the causing pathogen was not determined until 2006 [2]. The DHV-1 genome is 7691-nucleotide-long and encodes a polyprotein of 2250 amino acids that is proteolitically processed to the individual viral proteins. Sequence analysis has assigned DHV-1 to a new genus in the *Picornaviridae *family [3]. Picornaviruses are a large family of viruses that include a number of important human and animal pathogens, such as Enterovirus, Rhinovirus, Cardiovirus, Aphthovirus, Hepatovirus, Parechovirus, Erbovirus, Kobuvirus and Teschovirus [4]. Although these viruses have different host range and characteristics, they share common typical features, such us similar genome composition, genome structure and biological functions. The highly structured 5'-UTR of the picornavirus genome has been extensively characterized. The internal ribosome entry site (IRES) element located within this 5'-UTR directs internal initiation of viral protein synthesis in the infected cell [5].

The majority of host cell mRNAs are translated in a *cap*-dependent manner involving the recognition of their 5'-*cap *structure by the eIF4F complex [6]. The eIF4F complex comprises three proteins: the eIF4E, the cellular *cap*-binding factor; the eIF4A, an RNA helicase responsible for the ATP-dependent elimination of secondary structures near the 5'-*cap *of the mRNAs; and the eIF4G, which functions as a scaffold to bind several factors such as the eIF3, the poly(A)-binding protein (PABP), the eIF4E or the eIF4A. Subsequently to the eIF4F binding to the 5'-*cap *structure, the 43S pre-initiation complex is recruited to the mRNA, by its interaction with the eIF3, and the selected mRNAs are efficiently translated [7,8]. Initiation of translation is a major target for gene expression regulation [9-11] and viruses have evolved numerous unconventional mechanisms to preferentially recruit cellular translational machinery to the viral mRNA. Often, interactions of viral proteins with the components of the eIF4F complex and with the viral mRNA allow selective viral protein translation, blocking protein synthesis of the infected cell [12,13]. Translation initiation of picornaviruses proceeds by the direct interaction of the cellular machinery with the highly structured 5' IRES elements in the viral mRNAs. Structural insights coupled with biochemical studies have revealed that the IRES substitutes for the activities of some translation initiation factors. However, internal initiation strategies used by different members of this family differ in the requirement for eIF4F complex components. For instance, EMCV IRES recruits ribosomal machinery without the contribution of the cellular *cap*-binding protein eIF4E but requires active eIF2, eIF3, eIF4A and the central domain of eIF4G [14]. However, HCV IRES replaces the whole eIF4F complex and translation machinery is recruited by direct interaction of eIF3 and the viral mRNA [15-17].

According to their secondary structure, picornavirus IRES elements can be divided into four groups that display distinct biological properties [18,19]. The first group (class I) includes the IRES elements from entero- and rhinoviruses (e.g. poliovirus, PV) [20], while the second includes cardio- and aphthoviruses IRES elements (e.g. encephalomyocarditis virus, EMCV). The IRES element from hepatitis A virus (HAV) represents the third type of IRES [21,13], while the fourth group of picornavirus IRES elements has been recently described and includes porcine teschovirus-1 (PTV-1) Talfan strain, simian virus 2[22-24], porcine enterovirus-8 (PEV-8) [25], simian picornavirus 9 and avian encephalomyelitis virus (AEV) [26]. It has been reported that the IRES elements of this group are similar to HCV IRES in sequence, function and predicted secondary structure.

Computer-assisted analysis revealed that the 626-nt-long 5'-UTR region of the DHV-1 RNA genome folds into a compact IRES-like structure with some similarities with PTV-1 and HCV-like IRESes [27]. The prediction of the RNA structure indicates that it contains the stem-loop structures found in other type II picornaviruses but not the clover leaf structures typically found in type I picornaviruses. These data suggest the presence of an IRES element in the 5'-UTR of DHV-1 RNA that could direct viral protein synthesis. To confirm this prediction, we have examined if the 5'-UTR region of DHV-1 genome could direct translation initiation both in *in vivo *and *in vitro *assays and we have characterized the presence of accessory regulatory sequences and the requirement for eIF4F complex components.

## Results

### Conserved secondary structure elements in DHV-1 and HCV-like IRES RNAs

**Figure 1:**
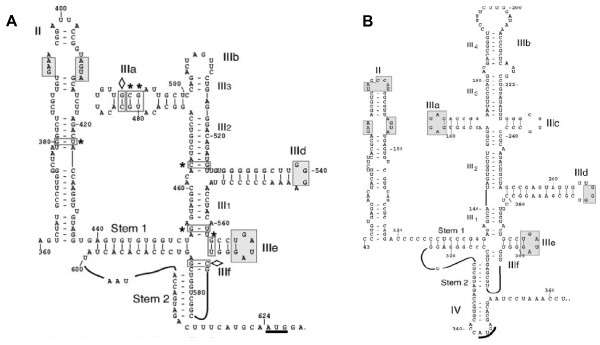
**Conserved secondary structure elements in DHV-1 5'-UTR and HCV-like IRES RNAs**. **(A) **Predicted secondary structure of the entire DHV-1 IRES. Domains are labeled according to the corresponding domains of the HCV IRES shown in **(B) **The structure was predicted by comparative sequence analysis and M-fold software to predict the most thermodynamically favourable structures. AUG triplets marked with a solid black bar represent the translation initiation site for the viral polyprotein. Lightly shaded rectangles indicate base pairs that are maintained despite sequence variation between types, strains, and isolates of the virus. Single-site substitutions that do not disrupt base pairing are indicated by asterisks, and base pairs maintained by paired covariant substitutions are indicated by lozenges. Unpaired bases that are conserved in the HCV IRES are indicated by gray shading.

Sequence analysis of the DHV-1 5'-UTR display a secondary structure with two major domains, II and III, which contain all the structural elements that have been described as crucial for internal translation initiation. The larger domain III consists on several branching high conserved hairpin stem-loops (III abcdef) (Figure 1A), which were also found in several members of the *Picornaviridae *family, such as the porcine teschovirus (PTV) and the avian encephalitis virus (AEV), and in some viruses from the *Flaviviridae *family, such as the classical swine fever virus (CSFV) and the hepatitis C virus (HCV) [28,2,11,29].

### DHV-1 5'-UTR is able to initiate cap-independent translation

**Figure 2:**
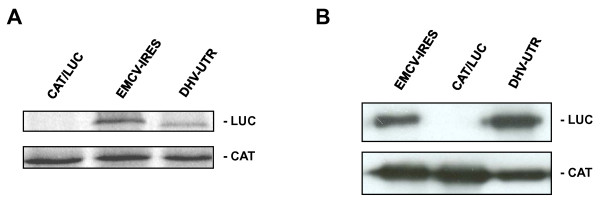
**The DHV 5'UTR initiates translation both *in vitro *and *in vivo***. **(A) **Dicistronic capped RNAs were *in vitro *generated from plasmid containing a cDNA insert corresponding to the DHV-1 5'-UTR (DHV UTR), pGEM-CAT/EMCV/LUC (EMCV-IRES) or pGEM-CAT/LUC (CAT/LUC). RNAs were individually added to Flexi rabbit reticulocyte lysate (RRL) system (Promega) and the *in vitro *translation of the reporter proteins was evaluated measuring ^35^S-methionine incorporation. **(B) **The dicistronic plasmids were transfected into vFT7-infected BHK-21 cells, and 24 h post-transfection, cell extracts were prepared and protein synthesis was analyzed by SDS-PAGE and immunoblotting to detect CAT and fLUC expression.

In order to evaluate the IRES activity of the 5'-UTR region of the DHV-1 genome, a dicistronic reporter plasmid was constructed by the insertion of a cDNA corresponding to the DHV-1 5'-UTR (nts 1 to 626) between two reporter gene sequences, the first encoding CAT protein and the second encoding fLUC (DHV UTR). Dicistronic plasmid pGEM-CAT/EMCV/LUC (EMCV-IRES), which contains the EMCV IRES, was used as a positive control for internal translation initiation activity, and the plasmid pGEM-CAT/LUC (CAT/LUC), which lacks any IRES sequence, was used as a negative control. The dicistronic plasmids were *in vitro *transcribed as indicated in Materials and Methods and the resultant RNAs were individually added to Flexi rabbit reticulocyte lysate (RRL) system (Promega). The *in vitro *translation of the reporter proteins was evaluated measuring ^35^S-methionine incorporation. *Cap*-dependent translation from the first ORF was determined by the level of CAT expression and internal initiation activity of the different sequences included between the two reporter genes was estimated by the accumulation of fLUC. All the mRNAs expressed CAT efficiently as expected (Figure 2A). Moreover, plasmids containing the EMCV IRES and the DHV-1 5'-UTR sequence in sense orientation allowed the efficient expression of fLUC (Figure 2A). These results suggest that the DHV-1 5'-UTR sequence is able to efficiently initiate cap-independent protein synthesis *in vitro*.

To confirm and extend the results from these *in vitro *assays, the same dicistronic plasmids were tested *in vivo *by transient-expression experiments in mammalian cells (Figure 2B). The dicistronic plasmids were transfected into vFT7-infected BHK-21 cells, and 20 h post-transfection, cell extracts were prepared and protein synthesis was analyzed by SDS-PAGE and immunoblotting to detect CAT and LUC expression. As expected, all plasmids expressed CAT efficiently. Moreover, the DHV-1 5'-UTR and the EMCV IRES containing plasmids also produced efficient fLUC accumulation. These results indicated that the putative DHV-1 IRES element is able to efficiently initiate protein synthesis *in vivo*.

### Stem1, Stem2 and domain IIIe are essential to the function of DHV-1 IRES

**Figure 3:**
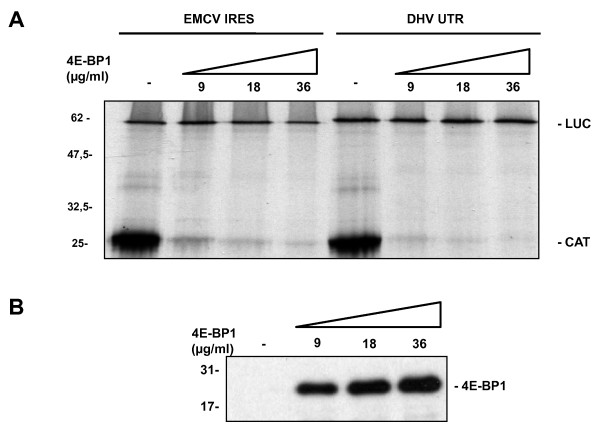
**Translation initiation of putative DHV-1 IRES is not inhibited by 4E-BP1**. The dicistronic capped RNAs indicated in the figure were added to Flexi RRL in the presence of increasing amounts of purified recombinant 4E-BP1 protein (0, 9, 18 and 36 μg/ml) and the *in vitro *translation of the reporter proteins was evaluated measuring ^35^S-methionine incorporation. **(B) **Western-blot analysis of the recombinant 4E-BP1 protein added in the different conditions.

**Figure 4:**
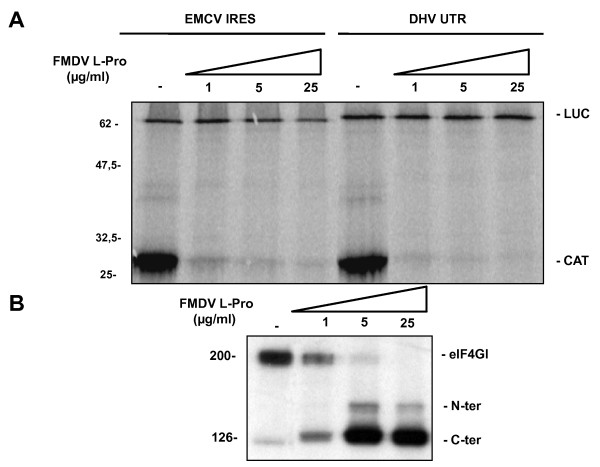
**The DHV-1 IRES does not require intact eIF4G**. **(A) **Flexi rabbit reticulocyte lysates were preincubated with or without recombinant FMDV L protease at a final concentration of 0, 1, 5, 25 μg/ml prior to the individual addition of the dicistronic capped RNAs indicated in the figure and translation of the reporter proteins was evaluated. **(B) **Western-blot showing the effect of recombinant FMDV L protease addition on eIF4GI.

Although there is only limited primary sequence relatedness among the 5'-UTRs of DHV-1, AEV and HCV, the structure of domain IIIe appears remarkably conserved among these viral IRESes [2,35,11]. It has been previously reported that domain IIIe is critical for the HCV, PTV-1 or AEV IRES-mediated initiation of translation. To evaluate the relevance of the putative IIIe region in DHV-1 IRES activity, the most important loop sequence within the IIIe region (GAUA) was mutated to AAAA (DHV IIIe mut) (Figure 3A) and the effect of the mutation on the translation initiation was evaluated. Mutant constructs were analyzed both in Flexi RRL (Figure 3) and in DF-1 cells (Figure 4). The results reveal that these mutations partially inhibited translation initiation both *in vitro *and *in vivo*, confirming the relevance of domain IIIe for DHV-1 IRES activity. Surprisingly, the reduction in the translational activity of the DHV-1 IRES upon disruption of the domain IIIe (50%) is moderate when compared with similar mutations in other type IV IRESes (90-95%).

Different experiments carried out with other picornaviruses, such as the HCV, PTV or AEV, showed that stem 1 and stem 2 within the IRES structure are crucial for translation initiation. The disruption of the base pair interactions in these regions seriously damages the ability to initiate translation. To analyze the role of the putative stems loops of the DHV-1 IRES, different mutations were introduced to disrupt the predicted base pairing of stem 1 (DHV stem1 mut) and stem 2 (DHV stem2 mut) (Figure 3A). The mutant constructs were analyzed by triplicate both in Flexi RRL (Figure 3) and in DF-1 cells (Figure 4). The results showed that both mutations resulted in a slight reduction in translation initiation ability of DHV-1 IRES both *in vitro *and *in vivo*. Moreover, the function of the IRES was partially recovered with the corresponding mutations that restore the structure (DHV stem1 mut' and DHV stem2 mut' respectively), confirming that the predicted pseudo-knot structures are formed *in vivo *and that they play a role in viral translation regulation. Once more, the reduction in the translational activity of the DHV-1 IRES upon disruption of these structures (50%) is moderate when compared with similar mutations in other type IV IRESes (90-95%).

### DHV-1 IRES activity is modulated by downstream coding sequences

**Figure 5:**
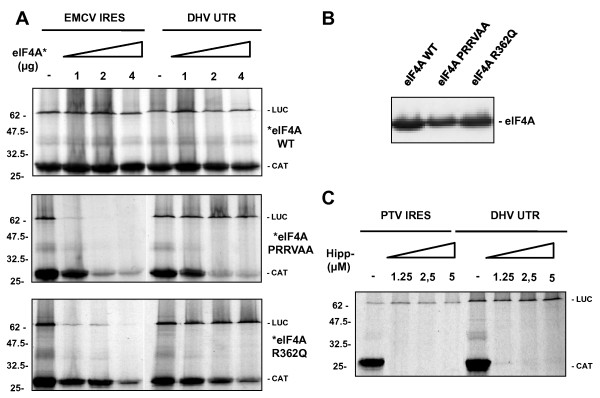
**DHV-1 IRES initiation does not require functional eIF4A**. **(A) **Increasing amounts of different dominant negative mutants were added to RRL prior to the individual addition of the dicistronic capped RNAs indicated in the figure and translation of the reporter proteins was evaluated. **(B) **Purification and expression efficiency of the different eIF4A mutants used in (A). **(C) **The *in vitro *translation efficiency of the different dicistronic constructs indicated in the figure was evaluated in the RRL in the presence of increasing amounts of hippuristanol.

To further characterize the presence of accessory regulatory sequences downstream of the 5'-UTR of the viral genome, 4 additional dicistronic reporter plasmids were constructed, containing DHV-1 5'-UTR and 10, 40 or 60 nucleotides of the coding region adjacent to the viral 5'-UTR (DHV-1 UTR+10nt, UTR+40nt, UTR+60nt, respectively) (Figure 5). Initiating AUG codon of the DHV-1 IRES was mutated to allow the expression of the luciferase from its own AUG. The dicistronic plasmids were *in vitro *transcribed and individually added to Flexi rabbit reticulocyte lysate (RRL) system (Promega). The translation of the reporter proteins was evaluated measuring ^35^S-methionine incorporation. *Cap*-dependent translation from the first ORF was determined by the level of CAT expression and internal initiation activity of the different sequences included between the two reporter genes was estimated by the accumulation of fLUC. All the constructs expressed CAT efficiently as expected but the efficiency of the DHV-1 UTR+10nt, UTR+40nt and UTR+60nt sequences was stronger than that of the DHV-1 UTR (Figure 5A). Moreover, the dicistronic plasmids were transfected into vFT7-infected BHK-21 cells and, in order to evaluate the *in vivo *internal initiation activity, the accumulation of fLUC protein expressed from the different constructs was determined and corrected by CAT expression levels. The experiments were performed by triplicate. As observed in the *in vitro *experiments, the translation initiation efficiency of DHV-1 UTR+10nt, UTR+40nt and UTR+60nt sequences was stronger than that of the DHV-1 UTR (Figure 5B). These results indicate the presence of accessory regulatory sequences downstream the DHV-1 putative IRES, which are not required for translation initiation but positively modulate the IRES activity.

### Characterization of the putative DHV-1 IRES requirement for eIF4F complex components

#### Translation initiation of DHV-1 IRES is not inhibited by 4E-BP1

**Figure 6:**
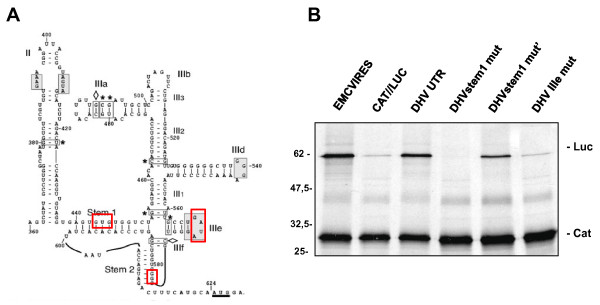
**Predicted domain IIIe and stem1 on DHV-1 5'-UTR are required for *in vitro *IRES activity**. **(A) **The position of the different mutations evaluated in Figures 7 and 8 is shown in red. **(B) **To evaluate the relevance of the predicted structures in DHV-IRES, a dicistronic plasmid, in which the most important loop sequence within the putative IIIe region (GAUA) is mutated to AAAA (DHV IIIe mut), was constructed. A different dicistronic plasmid containing mutations to disrupt the predicted base pairing of stem 1 (DHV stem1 mut) and the corresponding reverse mutant that restores the structure (DHV stem1 mut') were also constructed. Mutant constructs were *in vitro *transcribed analyzed in RRL and translation of the reporter proteins was evaluated.

Picornavirus IRES-mediated translation is independent of the cellular *cap*-binding protein eIF4E. In order to evaluate the putative DHV-1 IRES requirement for the eIF4E, the *in vitro *translation efficiency of the dicistronic constructs was evaluated in RRL as described upon addition of increasing amounts of purified recombinant 4E-BP1 protein. The eIF4E-binding proteins (4E-BPs) are a family of three small polypeptides that inhibit *cap*-dependent translation by binding to the eIF4E, obstructing its interaction with eIF4G [30]. However, the internal translation initiation on IRES elements is not affected in these conditions. As shown in Figure 6A, whereas the recombinant 4E-BP1 efficiently inhibited the *cap*-dependent CAT expression in RRL, the EMCV IRES-directed and DHV-1 5'-UTR-directed translation were unaffected.

#### The DHV-1 IRES does not require intact eIF4G

**Figure 7:**
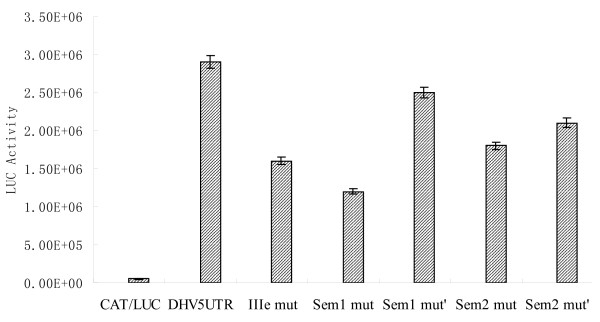
**Predicted domain IIIe, stem1 and stem2 on DHV-1 5'-UTR are required for *in vivo *IRES activity**. A dicistronic plasmid containing mutations to disrupt the predicted base pairing of stem 2 (DHV stem2 mut) and the corresponding reverse mutant that restores the structure (DHV stem2 mut') were constructed. *In vivo *translation efficiency of these new plasmids was evaluated, together with the plasmids depicted in Figure 7. Briefly, dicistronic plasmids were transfected into DF-1 cells and 48 hpt the accumulation of fLUC and CAT protein expressed from the different constructs was evaluated. fLUC expression was normalized by CAT expression levels.

According to the prediction of the 5'-UTR secondary structure, DHV-1 could be included among the type IV picornaviruses. Previous studies have shown that the type IV IRES elements (HCV-like IRESes) do not require the eIF4G activity to direct translation initiation and, consequently, that are insensitive to the eIF4G cleavage induced by different viral proteases, such as the foot-and-mouth disease virus (FMDV) L protease. The FMDV L protease efficiently cleaves the eukaryotic eIF4G resulting in a partial inhibition of the *cap*-dependent translation while the IRES-directed translation is not inhibited in these conditions and it can even be enhanced [31]. To confirm that the translation of DHV-1 5'-UTR is *cap*-independent and to examine its eIF4G dependence, the effect of foot-and-mouth disease virus (FMDV) L protease addition in the RRL system was evaluated. The dicistronic RNA constructs indicated in Figure 7A were individually added to FMDV-L treated or control Flexi RRL and the translation of the reporter proteins was evaluated. Whereas the *cap*-dependent CAT expression was efficiently inhibited, the EMCV-directed and the DHV-1 5'-UTR-directed translation were insensitive to FMDV L protease addition. Western blot analysis was performed to control the efficiency of the eIF4G cleavage by FMDV L protease (Figure 7B). These results indicate that the DHV IRES directs *cap*-independent internal initiation of translation and not requires an intact full-length eIF4G.

#### DHV-1 IRES initiation does not require functional eIF4A

**Figure 8:**
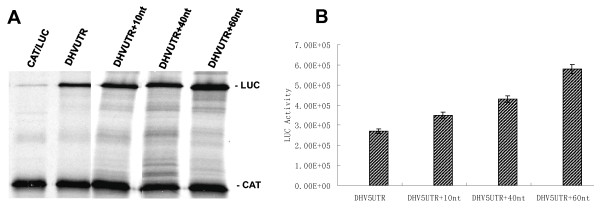
**DHV-1 IRES activity is modulated by downstream coding sequences**. **(A) **Four additional reporter plasmids were constructed containing DHV-1 5'-UTR and 10, 40 or 60 nucleotides, corresponding to the coding region adjacent to the viral 5'-UTR (DHV-1 UTR+10nt, UTR+40nt, UTR+60nt, respectively). Dicistronic plasmids were *in vitro *transcribed and capped RNAs were individually added to Flexi rabbit reticulocyte lysate (RRL) system (Promega) and the *in vitro *translation of the reporter proteins was evaluated measuring ^35^S-methionine incorporation. **(B) **Dicistronic plasmids were transfected into vFT7-infected BHK-21 cells and the accumulation of fLUC protein expressed from the different constructs was determined.

In order to evaluate the contribution of the remaining component of the eIF4F complex, the RNA helicase eIF4A, two different experimental *in vitro *approaches were used. Firstly, the effect on DHV-1 translation initiation of two different dominant negative mutants of eIF4A was evaluated. The dominant negative mutant PRRVAA contains a mutation in the conserved Ia region (PTRELA to PRRVAA) and is inactive in the ATP hydrolysis and the RNA unwinding activities [31]. The dominant negative mutant R362Q contains a mutation in the conserved arginine in position 362 (R362Q) of the C-terminal region HRIGRXXR, and exhibits drastically reduced RNA binding and RNA helicase activity [32]. The dicistronic RNAs indicated in Figure 8A were individually added to control Flexi RRL or eIF4A dominant negative mutant-containing RRL and translation of the reporter proteins was evaluated. The EMCV IRES, a class 2 IRES dependent on eIF4A activity, was used as negative control. Whereas the *cap*-dependent CAT expression and the EMCV-directed translation were efficiently inhibited, DHV-1 the IRES-directed translation was insensitive to eIF4A dominant negative mutant.

To further analyze the eIF4A requirement and confirm these results, *in vitro *experiments were carried out using increasing amounts of a small molecule inhibitor of eIF4A. The hippuristanol is a sterol isolated from the coral *Isis hippuris *and identified via a high throughput screening for general translation inhibitors. It has been shown to block the eIF4A-dependent translation by inhibiting its RNA binding, ATPase, and helicase activities by interaction with the C-terminal domain of the eIF4A [33,34]. The *in vitro *translation efficiency of the different dicistronic RNAs was evaluated in the RRL in the presence and in the absence of hippuristanol. In this experiment, PTV IRES, a class IV IRES which direct translation initiation independently of eIF4A, was used as positive control. C*ap*-dependent translation was efficiently inhibited by the eIF4A inhibitor, while PTV and DHV-1 IRES-directed translation were unaffected in these conditions (Figure 8C). These results indicate that, DHV-1 5'-UTR, as well as the class IV HCV-like IRESes, do not require functional eIF4A to initiate translation.

## Discussion

Picornavirus mRNAs are translated by an internal initiation mechanism, in which the ribosome enters directly at an internal site within the mRNA rather than scanning from the physical 5' end. So far, internal ribosome entry sites (IRESs) have been identified for all the *picornaviruses *as well as an increasing number of cellular mRNAs. The IRES elements in *picornaviruses *have been located within the 5' non-coding region, where the sequence and the RNA secondary structure are well conserved among viral serotypes in each genera. Based on sequence alignments, the genome of DHV-1 showed a typical *picornavirus *genetic organization and a putative class IV IRES element within DHV-1 5'-UTR. Therefore, DHV-1 should use similar *picornavirus*-like strategies for the initiation of translation. In this paper, we demonstrate, for the first time, that the DHV-1 5'-UTR has typical IRES activity, and that it can drive the translation of a downstream reporter gene both *in vitro *and *in vivo*. According to the prediction of the IRES structure, the DHV-1 IRES belongs to the type IV HCV-like IRESes, but it shares some common features with the IRES of viruses included in different groups.

Some picornaviruses, such as the AEV, contain HCV-like IRES elements organized in two major secondary structure domains, II and III [35,36,8], in which all the structural elements crucial for initiation of translation are located. Mutations within the putative loop of domain IIIe and stem1, 2 inhibited the DHV-1 IRES function. Moreover, the restoration of these structures partially recovered the activity of the DHV-1 IRES. These data provide strong evidence for the presence of the conserved domains II and III, common to other picornaviruses, within the DHV-1 5'-UTR and for the relevance of these regions to keep the normal internal initiation of translation of the DHV-1.

We have demonstrated that the sequences included within the DHV-1 Vp0 coding region contribute to the efficiency of internal initiation. The relevance of the coding sequences located immediately downstream of the IRESes has been previously described for other RNA viruses, such as the HCV or CSFV [22,24]. While most IRESs require only the 5'-non-translated region (5'-NTR) for full activity, the HCV or CSFV IRESes depend on the presence of protein-coding sequences downstream of the initiating AUG [37-39]. The minimal coding region elements required for high activity were exchanged between HCV and CSFV and the heterologous combinations were sufficiently active to rule out a highly specific functional interplay between the IRESes and the coding sequences [40]. Although nucleotide sequence of this region is different in CSFV and in HCV, they share common features such as being A rich and not highly structured. In fact, the efficiency of internal initiation of these chimeric constructs correlated with the degree of single-strandedness of the region around the initiation codon. According to secondary structure prediction, the DHV-1 coding region located immediately downstream of the IRES is not highly structured. This sensitivity to secondary structure around the initiation codon could be due to the fact that the eIF4F complex (which includes the eIF4A helicase as one of its subunits) is not required for translation initiation on these IRESs. However, the interaction of cellular proteins with the coding regions adjacent to the IRESes has been proposed as an alternative mechanisms that enhance viral translation, as it is the case of the NS1-associated protein 1 (NSAP1) and the HCV IRES [41], and cannot be discarded in the case of DHV-1.

Finally, we have characterized the DHV-1 IRES requirement for the eIF4F complex components and, as well as the EMCV and HCV-like IRESes, is insensitive to the FMDV L protease and the 4E-BP1 activities. These results indicate that the translation initiation takes places in the absence of fully functional eIF4E and eIF4G proteins. However, while the DHV-1 and HCV-like IRESes are not inhibited by hippuristanol or dominant negative forms of eIF4A, the EMCV IRES is strictly dependent on the eIF4A function [25]. Although the EMCV and DHV-1 IRESes share some common structural organization, they initiate translation by different mechanisms involving different cellular translation initiation factors. Moreover, as the DHV-1 and HCV share common structural motifs, they have similar requirement for the eIF4F complex components. Thus, it is likely that the translation initiation of DHV-1 could proceed similarly to PTV-1 and to other HCV-like IRES containing picornaviruses, by the direct recruitment of the 40S ribosomal subunit to the viral mRNA.

All together, these results demonstrate, for the first time, the existence of an IRES element within the highly structured 5'-UTR region of the DHV-1 genome. The DHV-1 IRES could be classified as type IV IRES attending to its secondary structure and biological functions and, consequently, it exhibits functional similarities with the HCV IRES. These new advances in the understanding of the DHV-1 IRES structure, function and interaction with translation-initiation factors provides a foundation for developing therapeutics to prevent the viral protein synthesis.

## Materials and methods

### RNA secondary structure prediction

5'-UTR sequence of DHV-1 ZJ strain (GenBank No. EF382778) was aligned with other DHV-1 isolates from GenBank using DNAstar (DNASTAR Inc.) software. Secondary structure elements were predicted in Mfold [42].

### Plasmid construction

DNA preparations were performed using standard methods as described in manufacturers' instructions. The reporter plasmids pGEM-CAT/EMC/LUC, containing the EMCV IRES cDNA, and pGEM-CAT/LUC were kindly offered by Ian Goodfellow (Imperial College, London, UK). These plasmids allow T7 promoter-directed expression of dicistronic mRNAs encoding chloramphenicol acetyl transferase (CAT) and firefly luciferase (LUC). For the construction of the DHV-1 5'-UTR-containing plasmid, a pair of primers (DHV-1 UTRF: 5'-GAGGATCCTTTGAAAGCGGGTGCATG-3'; DHV-1 UTRR: 5'-CGGGATTCTGCATGAAAG TCTACTGGT-3') were used to amplify the DHV-1 5'-UTR from wild DHV-1 strain (ZJ-V, GenBank No. EF382778). The purified PCR fragments were digested with BamHI, and then inserted into similarly digested and phosphatased pGEM-CAT/LUC between the two open reading frames (ORFs). The plasmid, containing the DHV-1 5'-UTR cDNA, was designated pGEM-CAT/DHV-1/LUC (Figure 1A). The further constructs containing the cDNA corresponding to the DHV-1 5'-UTR plus 10, 40, 60 nt of coding sequence were generated similarly using three pairs of specific primers and the resulting plasmids were termed pGEM-DHVUTR+10nt, pGEM-DHVUTR+40nt, pGEM-DHVUTR+60nt, respectively. Initiating AUG codon of the DHV-1 IRES was mutated to allow the expression of the luciferase from its own AUG.

### Mutagenesis of the DHV-1 cDNA

In order to introduce mutations in the predicted IIIe region, Quick-Change site-direct mutagenesis kit (Stratagene) was used in order to change the sequence in the loop region 570-573 from GAUA to AAAA. The plasmid pGEM-CAT/DHV-1UTR+60/LUC was used as the template with two specific primers: P1 (sense primer): 5'-CCTACACTGCCTAAAAGGGTCGCGGCTGGT-3'; P2 (antisense primer): 5'- CAGCCGCGACCCTTTTAGGCAGTGTAGGTT-3'. The resultant plasmid was named pGEMDHV IIIe mut. Similar strategies were used to introduce mutations within the stem sequences of the predicted pseudo-knots (termed Stem1-mut and Stem2-mut). For the generation of the Stem1-mutant, the nts 447-449 (TGT) were changed to CCC. The mutagenic primers were as follows: P3 (sense primer): 5'-TGTAGGTGAGTGCCCGGTCTAGAGTAGGC-3'; P4 (anti-sense primer): 5'-CTACTCTAGACCGGGCACTCACCTACAAC-3'. For the generation of the Stem2 mutant, the nts 604-605 (CC) were changed to GG. The mutagenic primers were as follows: P5 (sense primer): 5'-TGATAGGGTCGCCCCTGGTCGAGTCCCA-3'; P6 (antisense primer): 5'-GGACTCGACC AGGGGCGACCCTATCAGG-3'. The presence of all the expected mutations in the plasmids (pGEMDHV IIIe mut, pGEMDHV S1 mut and pGEMDHV S2 mut) was confirmed by DNA sequencing. Similar strategies were used to introduce the compensatory mutations in Stem1 mut' and Stem2 mut' constructs.

### *In vitro *translation reactions

For *in vitro *translation reactions, transcription of capped dicistronic mRNA was performed from the above plasmids, linearized with XhoI, using the Megascript transcription system (Ambion). Addition of the 7-mGTP cap 0 structure was performed using ScriptCap™m^7^G Capping System (Epicentre Biotechnologies) and mRNA was poly-adenylated using poly-A polymerase (PAP) following the suppliers' recommendations. *In vitro *transcribed mRNAs were added to the Flexi rabbit reticulocyte lysate (RRL) system (Promega) to a final concentration of 10 μg/ml. This concentration of RNA was previously determined to give a linear yield of the translated product over the time course of the experiment (90 min). In reactions that required the addition of either recombinant 4E-BP1, L-protease, the dominant negative mutant forms of eIF4A or hippuristanol, the RRL were pre-incubated with the recombinant proteins or with the eIF4A inhibitor at 30°C for 15 min prior to the addition of the different plasmids. After 90 min, the reactions were terminated by the addition of SDS-PAGE sample buffer and subsequently resolved on 12.5% polyacrylamide gels.

### Transient expression assays

The dicistronic reporter plasmids (1 μg) described above were transfected into BHK21 or DF-1 cells. Briefly, the plasmids were transfected into cells (35 mm dishes) previously infected with the recombinant vaccinia virus vTF7-3, which expresses T7 RNA polymerase, using Lipofectamine 2000 (Invitrogen) and Optimem (Gibco BRL). Cell lysates were prepared 24 h post-transfection and analyzed by SDS-PAGE and immunoblotting to determine CAT and LUC expression. Detection was achieved with anti-CAT (Sigma), anti-fLUC (Promega), peroxidase-labelled anti-rabbit (Amersham) or anti-goat (Dako Cytomation) antibodies respectively, using chemiluminescence reagents (Pierce). fLUC expression was also quantified using a firefly luciferase assay kit (Promega) with a luminometer according to the instructions of manufacturer.

### Recombinant proteins and reagents

Recombinant His-4E-BP1 was generously supplied by I. Goodfellow (Imperial College London). L-protease was kindly provided by T. Skern (University of Viena). Dominant negative eIF4A mutants were generously supplied by I. Goodfellow (Imperial College London). Hippuristanol was kindly provided by J. Pelletier (McGill University).

## Competing interests

The authors declare that they have no competing interests.

## Authors' contributions

GL and EY carried out the design of the study, performed analyses of data and edited the manuscript. CL carried out the plasmid construction and mutagenesis of the DHV-1 cDNA. ZC conducted some experiments (For example, transient expression assays) in the study. All authors read and approved the final manuscript.
